# Effects of high-flow oxygen therapy on patients with hypoxemia after extubation and predictors of reintubation: a retrospective study based on the MIMIC-IV database

**DOI:** 10.1186/s12890-021-01526-2

**Published:** 2021-05-13

**Authors:** Taotao Liu, Qinyu Zhao, Bin Du

**Affiliations:** 1 Department of Surgical Intensive Care Unit, Beijing Hospital, National Center of Gerontology, Institute of Geriatric Medicine, Chinese Academy of Medical Sciences, Beijing, 100730 China; 2 College of Engineering and Computer Science, Australian National University, Canberra, 2600 Australia; 3 Department of Medical Intensive Care Unit, State Key Laboratory of Complex Severe and Rare Diseases, Peking Union Medical College Hospital, Chinese Academy of Medical Science and Peking Union Medical College, 1 Shuai Fu Yuan, Beijing, 100730 China

**Keywords:** High-flow nasal cannula, Hypoxemia, Ventilator weaning, MIMIC

## Abstract

**Background:**

To investigate the indications for high-flow nasal cannula oxygen (HFNC) therapy in patients with hypoxemia during ventilator weaning and to explore the predictors of reintubation when treatment fails.

**Methods:**

Adult patients with hypoxemia weaning from mechanical ventilation were identified from the Medical Information Mart for Intensive Care IV (MIMIC-IV) database. The patients were assigned to the treatment group or control group according to whether they were receiving HFNC or non-invasive ventilation (NIV) after extubation. The 28-day mortality and 28-day reintubation rates were compared between the two groups after Propensity score matching (PSM). The predictor for reintubation was formulated according to the risk factors with the XGBoost algorithm. The areas under the receiver operating characteristic curve (AUC) was calculated for reintubation prediction according to values at 4 h after extubation, which was compared with the ratio of SpO_2_/FiO_2_ to respiratory rate (ROX index).

**Results:**

A total of 524,520 medical records were screened, and 801 patients with moderate or severe hypoxemia when undergoing mechanical ventilation weaning were included (100 < PaO2/FiO2 ≤ 300 mmHg), including 358 patients who received HFNC therapy after extubation in the treatment group. There were 315 patients with severe hypoxemia (100 < PaO2/FiO2 ≤ 200 mmHg) before extubation, and 190 patients remained in the treatment group with median oxygenation index 166[157,180] mmHg after PSM. There were no significant differences in the 28-day reintubation rate or 28-day mortality between the two groups with moderate or severe hypoxemia (all *P* > 0.05). Then HR/SpO_2_ was formulated as a predictor for 48-h reintubation according to the important features predicting weaning failure. According to values at 4 h after extubation, the AUC of HR/SpO_2_ was 0.657, which was larger than that of ROX index (0.583). When the HR/SpO_2_ reached 1.2 at 4 h after extubation, the specificity for 48-h reintubation prediction was 93%.

**Conclusions:**

The treatment effect of HFNC therapy is not inferior to that of NIV, even on patients with oxygenation index from 160 to 180 mmHg when weaning from ventilator. HR/SpO_2_ is more early and accurate in predicting HFNC failure than ROX index.

## Background

High-flow nasal cannula (HFNC) treatment can offer continuously higher gas flow with better heat and humidity than conventional oxygen [1]. It is also popular because of its easy application and good tolerability [2]. Several high-quality studies have shown that the treatment effect of HFNC on patients with hypoxemia or patients after surgery is not inferior to that of noninvasive ventilation (NIV) [3, 4]. However, both the indications for HFNC after early extubation in hypoxemic patients and the timing of reintubation when HFNC fails are unclear [5].

This retrospective study was designed based on the Medical Information Mart for Intensive Care IV (MIMIC-IV) database to investigate the indications for HFNC for patients with hypoxemia during ventilator weaning. A machine learning algorithm was used to explore the predictors of reintubation in these patients.

## Methods

### Patients

The patients were identified in the MIMIC-IV database from 2008 to 2019. The inclusion criteria were as follows: hypoxemia 4 h before extubation (100 < PaO_2_/FiO_2_ ≤ 300 mmHg); over 18 years old; with or without hypercapnia; and received continuous or intermittent HFNC or NIV after extubation. The exclusion criteria were as follows: tracheotomy; accidental extubation; and received both HFNC and NIV after extubation.

### Source of data and ethics approval

This retrospective study was conducted based on a large critical care database named Medical Information Mart for Intensive Care IV [6]. This database is an updated version of MIMIC-III with pre-existing institutional review board approval. A number of improvements have been made, including simplifying the structure, adding new data elements, and improving the usability of previous data elements. Currently, the MIMIC-IV contains comprehensive and high-quality data of patients admitted to intensive care units (ICUs) at the Beth Israel Deaconess Medical Center between 2008 and 2019 (inclusive). One author (QZ) obtained access to the database and was responsible for data extraction.

### Study design

The treatment group received continuous or intermittent HFNC after extubation, and the control group received continuous or intermittent NIV after extubation.

The following data were recorded: age, sex, body mass index (BMI), comorbidities, simplified acute physiology scoring II (SAPS-II) score at ICU admission, duration of mechanical ventilation, reintubation rate, mortality, length of ICU stay, length of hospital stay and duration before reintubation.

Physiological parameters and arterial blood gas (ABG) from 4 h before weaning to 48 h after extubation were collected. Average values for each patient per four hours were assessed, and the median value and interquartile ranges (IQRs) in the two groups were plotted. The 28-day mortality of patients who received reintubation within 48 h after extubation was compared with that of patients who received reintubation 48 h after extubation.

### Statistical analysis

Variables with normal distributions are presented as the means (SD) and were compared with independent samples *t* tests. Nonnormally distributed variables are expressed as medians and IQRs, which were compared with the Mann–Whitney U test. Categorical variables are described as percentages and were compared by using a chi-square test. A Kaplan–Meier curve was drawn to evaluate the time from extubation to reintubation, and a log-rank test was used to compare the differences in times between the two groups.

Above risk factors for reintubation were included for propensity score matching (PSM): age, gender, BMI, SAPS-II, comorbidities, heart rate, respiratory rate, mean blood pressure, pH, PaO_2_, PaCO_2_, PaO_2_/FiO_2_, SpO_2_ and ventilation duration before extubation. Multivariate Imputation by Chained Equations was used to impute missing values, followed by the development of a multivariate logistic regression model to estimate the patient’s propensity scores for HFNC treatment [7]. One-to-one nearest neighbour matching with a caliper width of 0.1 was applied in the present study [8]. Statistical testing was performed to evaluate the effectiveness of PSM. The duration before reintubation, 28-day mortality, and 48-h and 28-day reintubation rates were compared based on matched data. Additionally, subgroup analyses were separately performed on patients with moderate and severe hypoxemia. PSM was applied to each subgroup, and outcomes were compared based on the matched data.

The risk factors for reintubation were analysed by a machine learning algorithm. The extreme gradient boosting (XGBoost) model [9], an advanced ensemble learning algorithm, was developed to predict 48-hour reintubation risk based on the baseline variables. Feature importance was assessed by using the SHapley Additive exPlanations (SHAP) values [10]. Features were sorted according to the mean value of absolute SHAP values. Then, predictors were developed manually based on the baseline values of most important features. The areas under the receiver operating characteristic curve (AUCs) of the predictors to predict 48-hour reintubation were calculated and compared with the rapid shallow breathing index (RSBI) and the ratio of SpO_2_/FiO_2_ to respiratory rate (ROX index).

All statistical analyses were performed with R (version 3.6.1), and *p* < 0.05 was considered statistically significant.

## Results

### Propensity score adjusted and matched outcomes

**Fig. 1:**
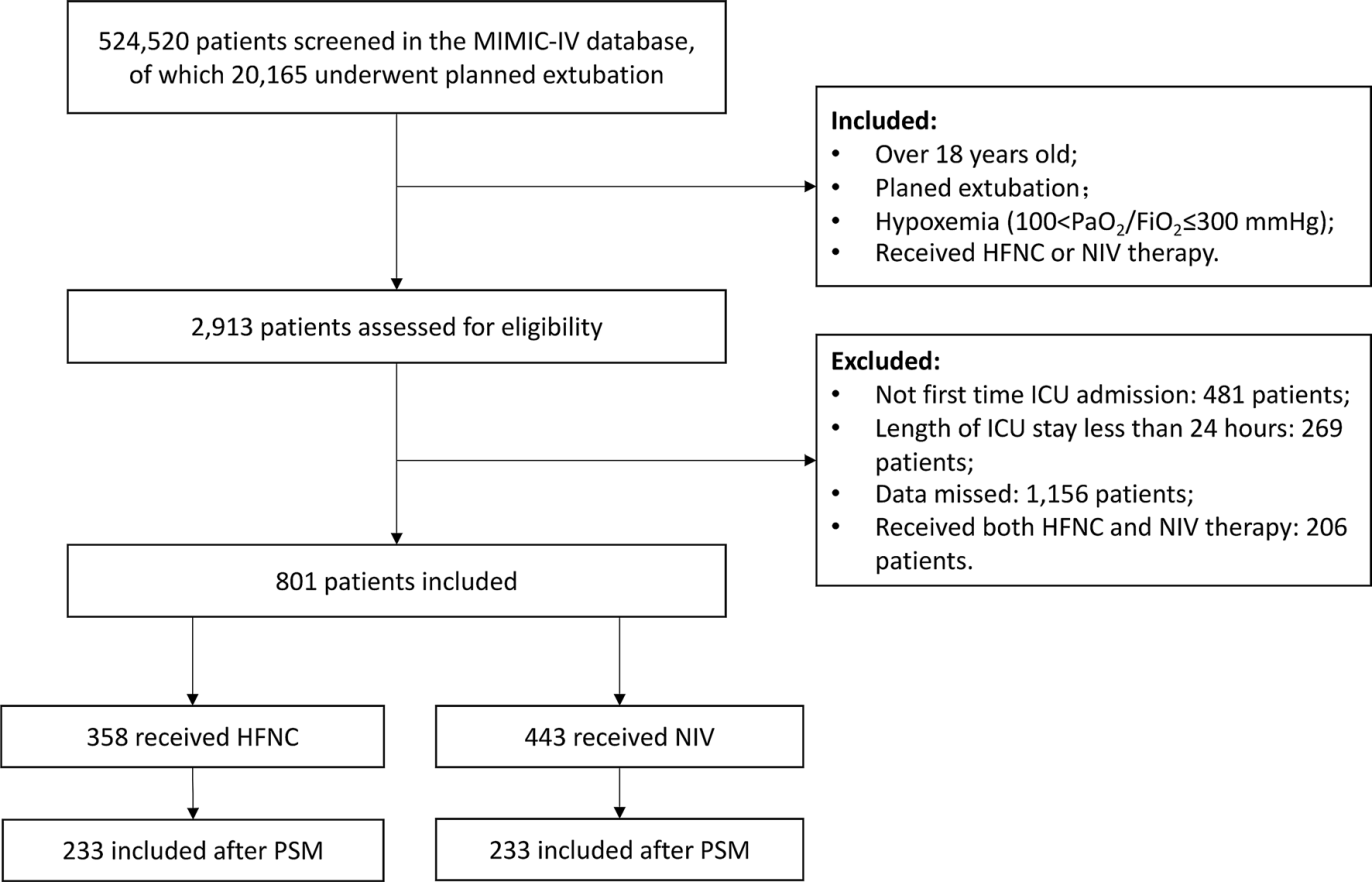
Flow chart of the study

A total of 524520 medical records were screened, including 20165 patients with planned extubation. Finally, 801 patients with moderate and severe hypoxemia when mechanical ventilation weaning was included (100<PaO2/FiO2≤300 mmHg), and 358 patients received HFNC therapy after extubation in the treatment group. There were 233 patients remained in the treatment group with median oxygenation index 209[164,253] mmHg after PSM (Fig. 1). There were no significant differences in age, sex, BMI, SPAS-II score, comorbidities, duration of mechanical ventilation or physiological parameters before weaning between the 2 groups (all *P*>0.05).

There were no significant differences in the 28-day reintubation rate (4.29% vs. 5.15%, *P*=0.827) or 28-day mortality (4.29% vs. 5.15%, *P*=0.827) between the two groups. The 48-hour reintubation rate in the treatment group was lower than that in the control group (8.58% vs. 15.88%, *P*=0.024).

There were 315 patients with severe hypoxemia (100<PaO2/FiO2≤200 mmHg) before extubation, and 190 patients remained in the treatment group with median oxygenation index 166[157,180] mmHg after PSM. There were no significant differences in the 48-hour reintubation rate, 28-day reintubation rate or 28-day mortality between the 2 groups (all *P*>0.05).

There were 486 patients with moderate hypoxemia (200<PaO2/FiO2≤300 mmHg) before extubation, and 304 patients remained in the treatment group with median oxygenation index 238[214,267] mmHg after PSM. There were no significant differences in the 48-hour reintubation rate, 28-day reintubation rate or 28-day mortality between the 2 groups (all *P*>0.05).

**Fig. 2:**
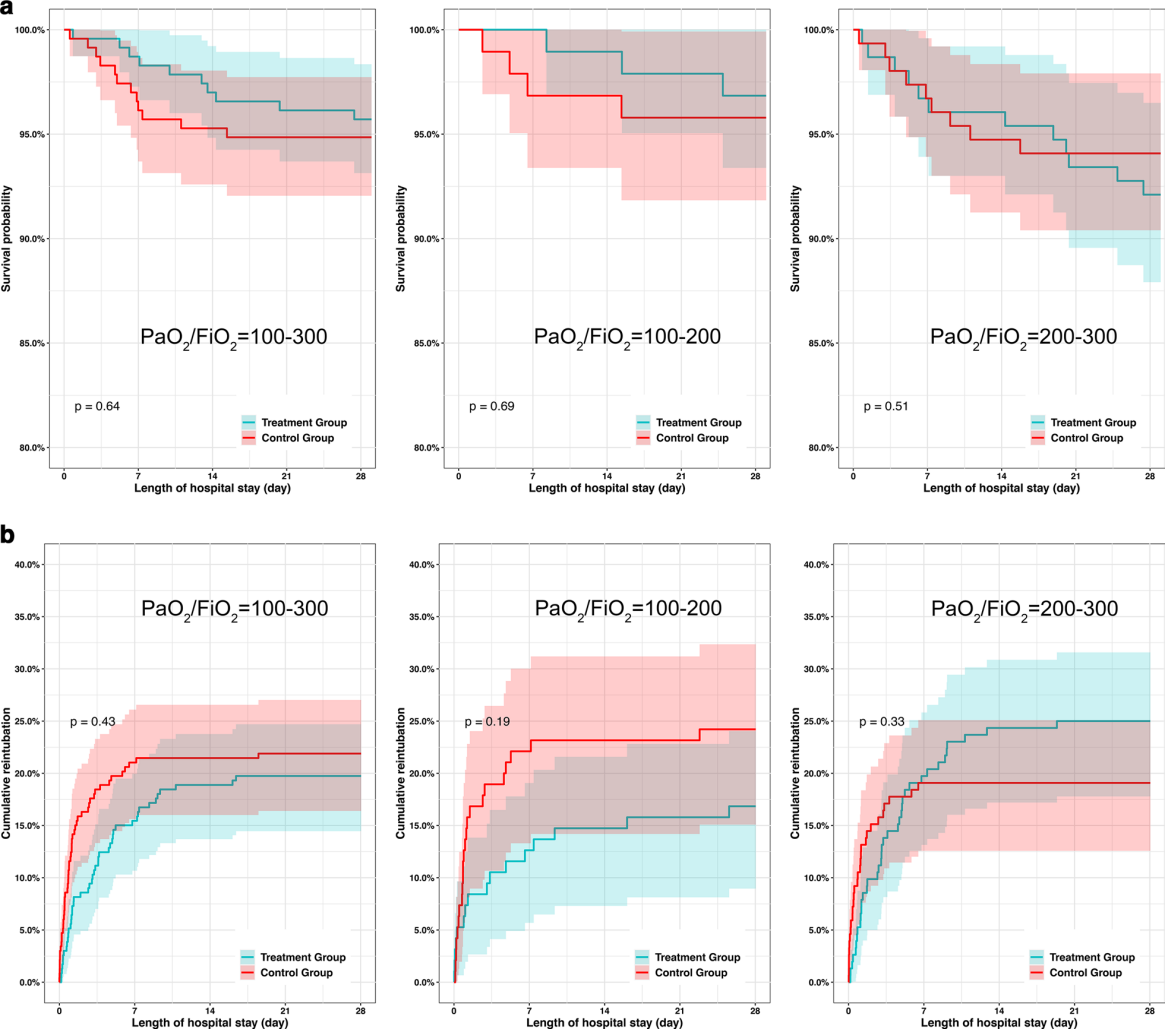
Survival curve and cumulative reintubation curve of patients with different severities of hypoxemia after PSM. **a** Survival curve of patients with different severities of hypoxemia after PSM. **b** Cumulative reintubation curve of patients with different severities of hypoxemia after PSM

Both the length of stay in the ICU and in the hospital in the treatment group were longer than those in the control group (6.36 vs. 4.72 days, *P*<0.001 and 12.62 vs. 10.93 days, *P*=0.001). The duration before reintubation in the treatment group was longer than that in the control group (73.28 vs. 21.52 hours, *P*=0.001) (Table 1 and Fig. 2).

**Table 1:** The baseline data and prognosis of patients with hypoxemia of different severities in the two groups after PSM

	100 < PaO_2_/FiO_2_ ≤ 300 n = 801				100 < PaO_2_/FiO_2_ ≤ 200 n = 315				200 < PaO_2_/FiO_2_ ≤ 300 n = 486			
	After PSM n = 466	Treatment group n = 233	Control group n = 233	*P* value	After PSM n = 190	Treatment group n = 95	Control group n = 95	*P* value	After PSM l n = 304	Treatment group n = 152	Control group n = 152	*P*
Age, median [Q1, Q3]	69.38[61.00, 77.59]	68.74[59.90, 77.81]	69.80[61.48, 76.32]	0.850	70.06[60.32, 78.09]	69.91[60.55, 77.58]	70.80[59.47, 78.50]	0.638	68.09[59.57, 75.49]	66.79[58.59, 75.85]	68.71[60.21, 75.22]	0.391
Male, n (%)	322(69.10)	158(67.81)	164(70.39)	0.616	126(66.32)	62(65.26)	64(67.37)	0.878	195(64.14)	92(60.53)	103(67.76)	0.232
BMI, mean (SD)	31.93(6.56)	32.04(6.60)	31.81(6.53)	0.708	33.85(6.47)	33.38(6.34)	34.34(6.61)	0.322	31.51(7.33)	31.09(6.93)	31.94(7.72)	0.330
Baseline disease												
Hypertension, n (%)	316(67.81)	157(67.38)	159(68.24)	0.921	123(64.74)	59(62.11)	64(67.37)	0.544	188(61.84)	92(60.53)	96(63.16)	0.723
Diabetes mellitus, n (%)	88(18.88)	47(20.17)	41(17.60)	0.554	30(15.79)	15(15.79)	15(15.79)	1.000	57(18.75)	32(21.05)	25(16.45)	0.378
COPD, n (%)	52(11.16)	29(12.45)	23(9.87)	0.462	17(8.95)	12(12.63)	5(5.26)	0.127	35(11.51)	18(11.84)	17(11.18)	1.000
Congestive heart failure, n (%)	133(28.54)	62(26.61)	71(30.47)	0.412	51(26.84)	20(21.05)	31(32.63)	0.102	79(25.99)	39(25.66)	40(26.32)	1.000
Myocardial infarction, n (%)	54(11.59)	28(12.02)	26(11.16)	0.885	25(13.16)	11(11.58)	14(14.74)	0.668	36(11.84)	19(12.50)	17(11.18)	0.859
Chronic kidney disease, n (%)	96(20.60)	51(21.89)	45(19.31)	0.567	35(18.42)	15(15.79)	20(21.05)	0.454	60(19.74)	33(21.71)	27(17.76)	0.471
Leukaemia, n (%)	3(0.64)	1(0.43)	2(0.86)	1.000	6(3.16)	2(2.11)	4(4.21)	0.682	3(0.99)	1(0.66)	2(1.32)	1.000
Strokes, n (%)	20(4.29)	11(4.72)	9(3.86)	0.819	5(2.63)	2(2.11)	3(3.16)	1.000	20(6.58)	13(8.55)	7(4.61)	0.247
Cancer, n (%)	48(10.30)	25(10.73)	23(9.87)	0.879	25(13.16)	16(16.84)	9(9.47)	0.198	33(10.86)	15(9.87)	18(11.84)	0.712
Liver disease, n (%)	32(6.87)	14(6.01)	18(7.73)	0.583	12(6.32)	9(9.47)	3(3.16)	0.136	32(10.53)	20(13.16)	12(7.89)	0.191
SAPS-II at admission, mean (SD)	42.99(12.44)	43.00(12.96)	42.97(11.92)	0.979	43.17(13.09)	43.42(13.60)	42.93(12.62)	0.795	42.61(12.97)	43.12(13.54)	42.09(12.39)	0.491
Duration before extubation, median [Q1,Q3], hours	20.77[6.89, 65.71]	22.00[7.32, 73.27]	19.50[6.12, 48.85]	0.136	21.73[6.68, 57.68]	24.00[6.73, 68.37]	20.47[6.76, 47.30]	0.304	19.78[6.91, 81.10]	21.99[7.24, 108.54]	18.08[6.35, 46.92]	0.133
Physiological variables before extubation 4 h												
Heart rate, mean (SD)	83.15(13.84)	83.74(13.97)	82.55(13.72)	0.354	82.93(13.39)	82.72(12.30)	83.14(14.47)	0.832	83.94(13.47)	84.72(14.41)	83.17(12.47)	0.316
Respiratory rate, mean (SD)	18.99(3.95)	19.07(3.90)	18.91(4.00)	0.669	19.37(3.97)	19.40(4.06)	19.35(3.90)	0.938	18.81(3.97)	18.90(4.09)	18.73(3.86)	0.701
Tidal volume, mean (SD)	487.80(125.50)	493.81(127.26)	481.81(123.75)	0.337	504.61(134.40)	521.13(139.12)	487.07(127.73)	0.101	487.71(122.32)	487.63(124.30)	487.79(120.82)	0.991
MBP, mean (SD)	77.51(11.00)	77.80(11.62)	77.22(10.35)	0.570	77.91(10.51)	78.35(10.40)	77.46(10.65)	0.562	78.46(12.22)	78.83(13.21)	78.08(11.16)	0.591
pH, mean (SD)	7.40(0.05)	7.40(0.05)	7.39(0.05)	0.366	7.40(0.06)	7.40(0.06)	7.40(0.05)	0.358	7.39(0.05)	7.39(0.05)	7.39(0.05)	0.812
PaO_2,_ median [Q1, Q3]	100.00[84.00, 115.00]	97.75[83.00, 114.00]	101.50[86.00, 118.00]	0.208	84.42[76.25, 95.00]	84.50[78.75, 95.00]	84.33[76.00, 95.25]	0.924	109.00[98.88, 125.63]	107.00[95.38, 122.56]	110[100.50, 130.63]	0.340
PaCO_2,_ mean (SD)	41.01(6.96)	40.75(6.57)	41.26(7.34)	0.433	40.70[6.56]	40.61[6.12]	40.79[7.01]	0.852	41.08(6.65)	40.78(6.98)	41.37(6.32)	0.441
SpO2, median [Q1, Q3]	97.50[95.83, 98.75]	97.25[95.80, 98.75]	97.50[96.00, 99.00]	0.397	96.06[94.68, 97.79]	95.75[94.50, 97.52]	96.50[94.90, 98.20]	0.152	98.00[96.67, 99.25]	97.75[96.75, 99.16]	98.25[96.50, 99.25]	0.412
PaO_2_/FiO_2_, median [Q1,Q3]	211.79[171.42, 253.23]	209.00[164.00, 253.62]	213.00[179.33, 253.06]	0.253	169.46[155.08, 182.83]	166.67[157.44, 180.60]	171.33[153.44, 187.56]	0.283	242.00[217.50, 270.23]	238.46[214.00, 267.34]	248.04[222.00, 273.98]	0.209
Reintubation 48 h, n (%)	57(12.23)	20(8.58)	37(15.88)	0.024	24(12.63)	8(8.42)	16(16.84)	0.126	37(12.17)	15(9.87)	22(14.47)	0.293
Reintubation 28 days, n (%)	97(20.82)	46(19.74)	51(21.89)	0.648	39(20.53)	16(16.84)	23(24.21)	0.281	67(22.04)	38(25.00)	29(19.08)	0.268
Mortality 28 days, n (%)	22(4.72)	10(4.29)	12(5.15)	0.827	7(3.68)	3(3.16)	4(4.21)	1.000	21(6.91)	12(7.89)	9(5.92)	0.651
Duration before reintubation, median [Q1, Q3], hours	28.65[11.57, 90.78]	73.28[21.63, 124.15]	21.52[8.84, 56.85]	0.001	25.03[9.04, 113.43]	52.22[5.96, 163.10]	21.72[10.88, 66.22]	0.424	38.55[12.12, 111.62]	73.66[27.39, 133.14]	19.70[4.62, 40.63]	0.001
LOS in hospital, median [Q1, Q3]	11.54[7.18, 17.75]	12.62[7.65, 20.61]	10.93[6.83, 15.82]	0.001	11.87[7.65, 16.61]	12.80[7.79, 19.23]	11.28[7.48, 15.43]	0.102	12.01[7.02, 19.90]	14.59[8.68, 25.02]	10.12[6.22, 16.72]	< 0.001
LOS in ICU, median [Q1, Q3]	5.55[3.09, 11.14]	6.36[3.85, 13.59]	4.72[2.27, 9.70]	< 0.001	5.39[3.10, 10.93]	6.22[3.82, 12.69]	4.80[2.30, 9.39]	0.026	6.19[3.12, 13.14]	7.43[4.19, 15.89]	4.25[2.23, 9.47]	< 0.001

The 28-day mortality of patients with reintubation 48 hours after extubation was not higher than that within 48 hours in either the treatment group or the control group (23.08% vs. 10.00%, *P*=0.206 and 19.23% vs. 12.73%, *P*=0.509) (Table 2).

**Table 2:** The baseline data and prognosis of patients who received reintubation within 48 h of and 48 h after extubation in the two groups

	Treatment group n = 358				Control group n = 443			
	All reintubations n = 79	Within 48 h n = 40	48 h after n = 39	*P* value	All reintubations n = 81	Within 48 h n = 55	48 h after n = 26	*P* value
Age, median[Q1, Q3]	67.68[57.02, 78.00]	64.47[49.57, 77.97]	68.83[62.52, 77.59]	0.202	71.82[62.27, 78.93]	71.52[60.69, 78.69]	73.40[64.81, 78.74]	0.485
Male, n (%)	59(74.68)	31(77.50)	28(71.79)	0.746	48(59.26)	29(52.73)	19(73.08)	0.134
BMI, mean (SD)	29.65(5.87)	28.99(5.72)	30.38(6.02)	0.314	32.60(9.00)	31.12(8.34)	35.67(9.71)	0.051
Baseline disease								
Hypertension, n (%)	41(51.90)	18(45.00)	23(58.97)	0.309	54(66.67)	33(60.00)	21(80.77)	0.110
Diabetes mellitus, n (%)	12(15.19)	7(17.50)	5(12.82)	0.790	13(16.05)	7(12.73)	6(23.08)	0.331
COPD, n (%)	5(6.33)	3(7.50)	2(5.13)	1.000	9(11.11)	5(9.09)	4(15.38)	0.458
Congestive heart failure, n (%)	23(29.11)	10(25.00)	13(33.33)	0.570	29(35.80)	17(30.91)	12(46.15)	0.277
Myocardial infarction, n (%)	6(7.59)	4(10.00)	2(5.13)	0.675	14(17.28)	9(16.36)	5(19.23)	0.760
Chronic kidney disease, n (%)	17(21.52)	5(12.50)	12(30.77)	0.089	21(25.93)	10(18.18)	11(42.31)	0.041
Leukaemia, n (%)	1(1.27)	0	1(2.56)	0.494	2(2.47)	1(1.82)	1(3.85)	0.542
Strokes, n (%)	8(10.13)	1(2.50)	7(17.95)	0.029	6(7.41)	4(7.27)	2(7.69)	1.000
Cancer, n (%)	10(12.66)	6(15.00)	4(10.26)	0.737	16(19.75)	9(16.36)	7(26.92)	0.415
Liver disease, n (%)	12(15.19)	7(17.50)	5(12.82)	0.790	14(17.28)	8(14.55)	6(23.08)	0.360
SAPS-II at admission, mean (SD)	44.32(13.08)	43.30(10.86)	45.36(15.09)	0.490	47.20(13.72)	46.73(13.48)	48.19(14.45)	0.665
Duration before extubation, median [Q1,Q3], hours	61.50[20.33, 125.27]	53.92[15.14, 110.82]	67.35[22.86, 138.07]	0.364	38.90[20.25, 131.67]	40.83[23.53, 128.29]	28.46[17.63, 127.40]	0.413
Physiological variables before extubation 4 h								
Heart rate, mean (SD)	87.28(15.55)	89.67(16.07)	84.84(14.80)	0.168	86.00(16.39)	87.03(14.03)	83.81(20.67)	0.476
Respiratory rate, mean (SD)	19.10(4.67)	19.41(4.09)	18.78(5.23)	0.556	19.73(4.17)	19.70(4.42)	19.79(3.64)	0.920
Tidal volume, mean (SD)	534.45(132.46)	527.92(146.70)	539.43(122.28)	0.734	454.31(111.36)	448.81(112.36)	466.35(110.87)	0.553
MBP, mean (SD)	79.62(12.93)	81.10(13.91)	78.10(11.82)	0.304	77.45(10.11)	77.61(10.55)	77.13(9.31)	0.836
pH, mean (SD)	7.41(0.07)	7.39(0.07)	7.42(0.06)	0.068	7.39(0.05)	7.39(0.06)	7.38(0.05)	0.831
PaO_2,_ median [Q1, Q3]	91.00[82.00, 107.00]	89.25[83.50, 104.62]	95.50[81.17, 110.50]	0.444	96.00[87.00, 108.00]	95.00[85.75, 106.25]	98.25[89.54, 115.00]	0.347
PaCO_2,_ mean (SD)	39.76(6.76)	39.52(6.60)	40.01(6.99)	0.751	44.79(10.47)	45.58(11.74)	43.10(7.01)	0.240
SpO2, median [Q1, Q3]	97.00[95.54, 98.69]	96.50[95.00, 98.29]	97.25[96.38, 98.88]	0.088	97.25[95.50, 98.60]	97.80[95.75, 98.78]	96.50[95.56, 97.93]	0.172
PaO_2_/FiO_2_, median [Q1,Q3]	209.00[175.50, 248.62]	208.00[170.00, 229.86]	217.50[189.75, 254.30]	0.233	220.00[187.50, 252.52]	213.00[186.50, 254.32]	221.88[192.12, 249.65]	0.712
Mortality 28 days, n (%)	13(16.46)	4(10.00)	9(23.08)	0.206	12(14.81)	7(12.73)	5(19.23)	0.509

### Features and predictors of HFNC failure

**Fig. 3:**
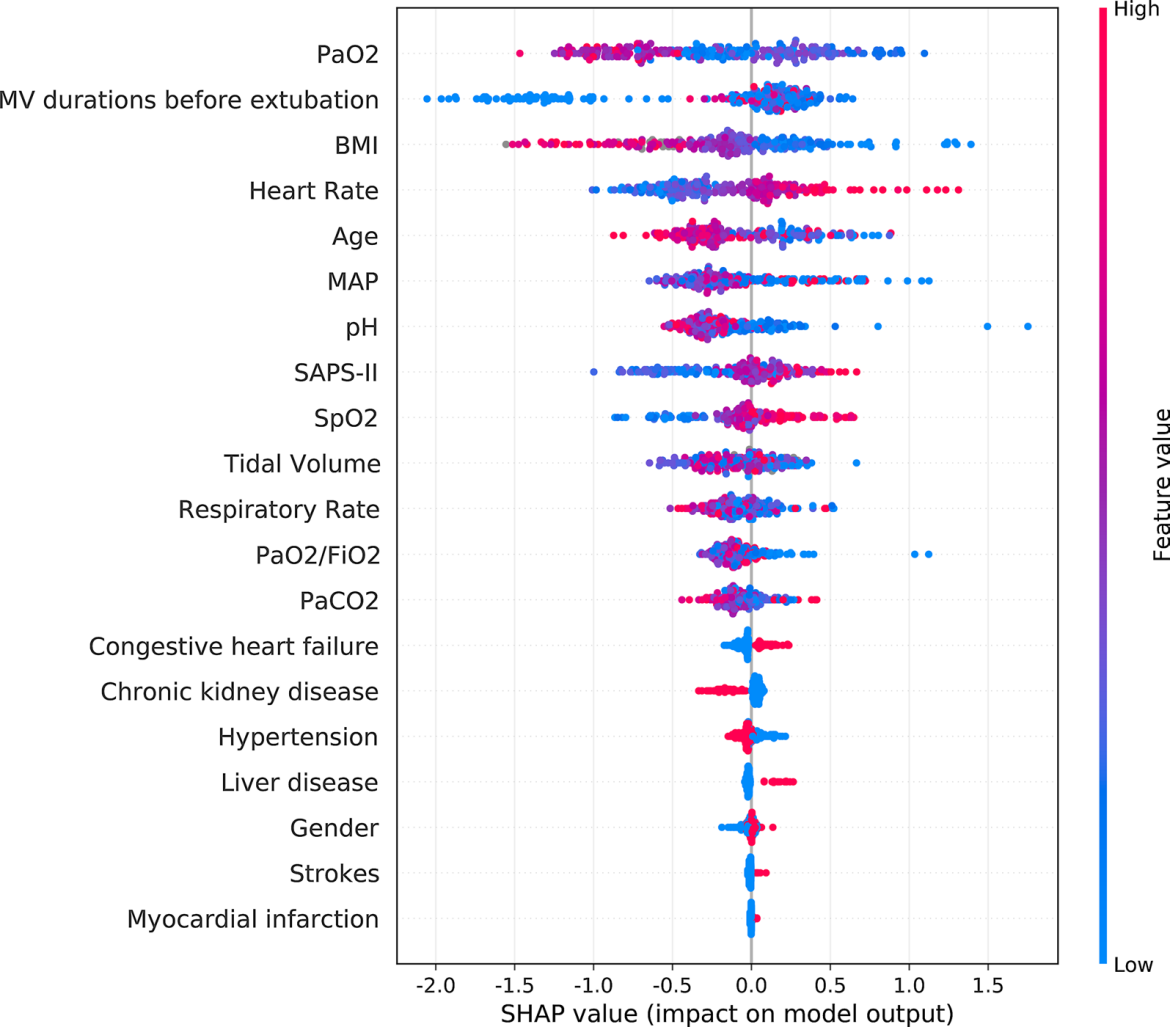
Important features of the machine learning XGBoost model in reintubation prediction

**Fig. 4:**
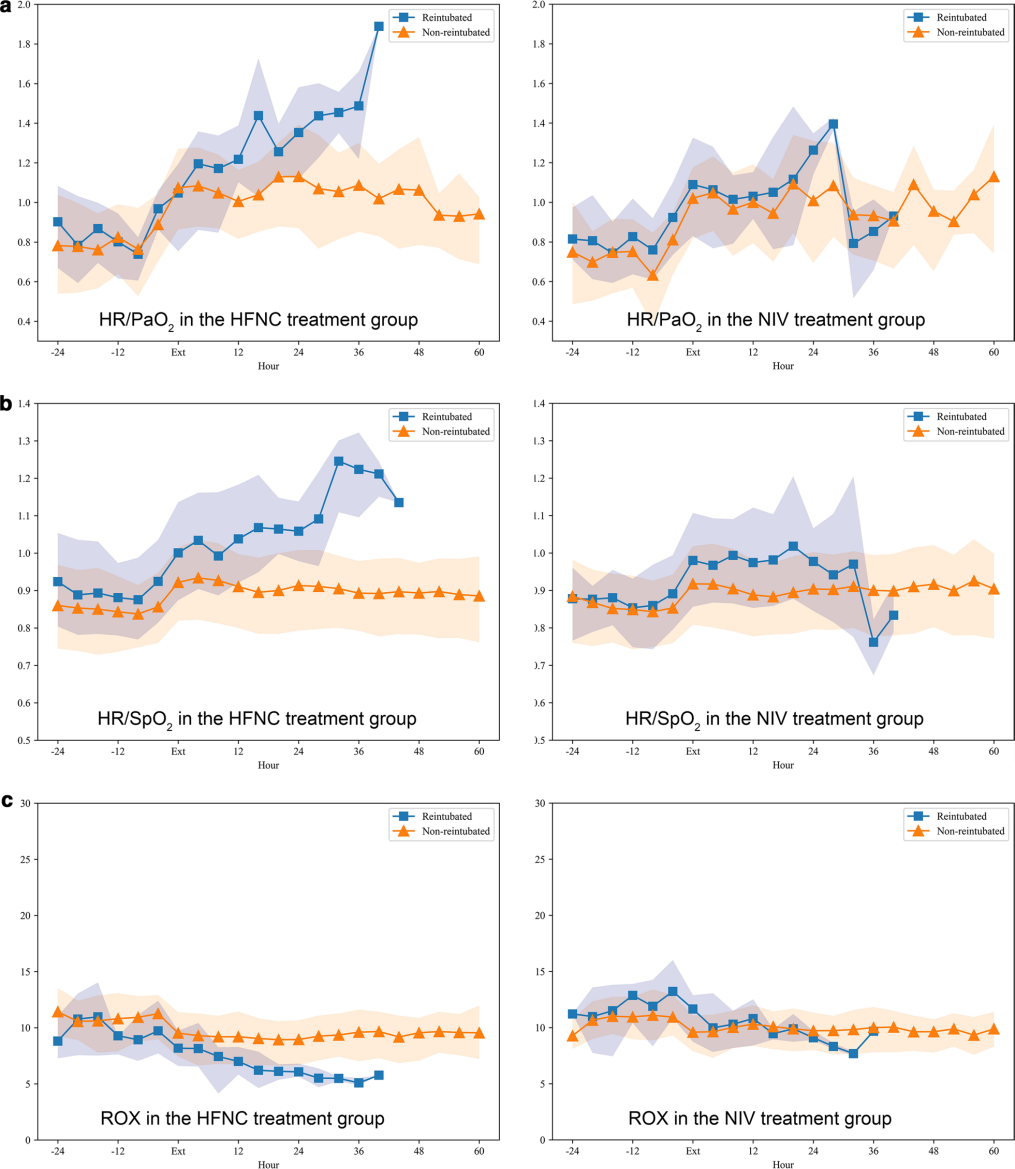
Changes in HR/PaO_2_, HR/SpO_2_ and the ROX index in patients who received reintubation within 48 h in the two groups. **a** HR/PaO_2_; **b** HR/SpO_2_; and **c** the ROX index

**Fig. 5:**
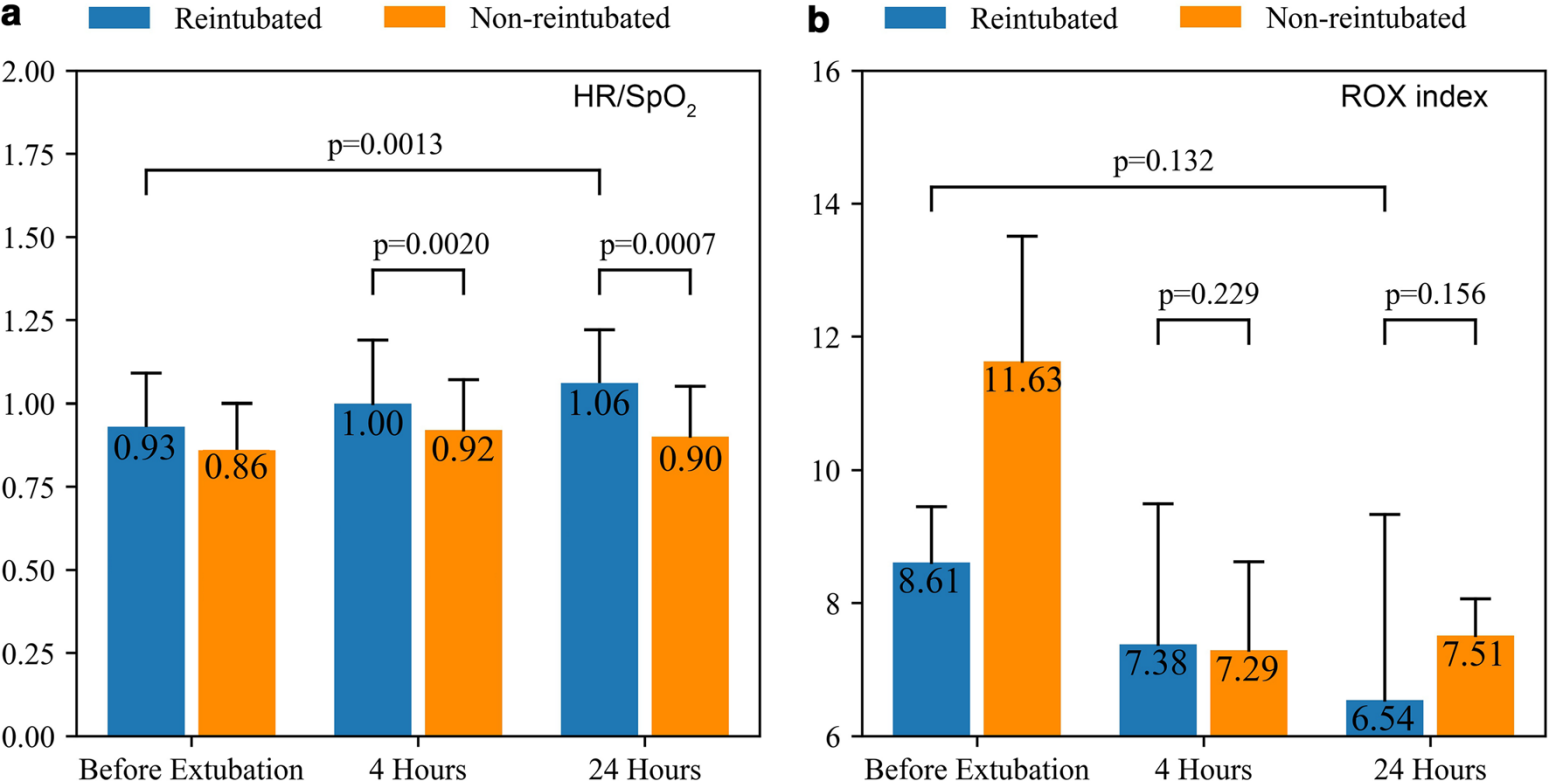
Values of HR/SpO_2_ and the ROX index at 4 and 24 h after extubation in two groups. **a** HR/SpO_2_; and **b** the ROX index

The important features predicting weaning failure were PaO_2_, duration before extubation, heart rate, BMI, age, mean blood pressure, pH, SAPS-II, SpO_2_, tidal volume and respiratory rate (Fig. 3). Thus HR/PaO_2_ and HR/SpO_2_ were calculated manually based on the above important features. There was a significant difference of HR/SpO_2_ at 4 hours after extubation between patients weaning failed and successfully (1.00 vs. 0.92, *P*< 0.05), and no significant difference of ROX index at the same time (7.38 vs. 7.29, *P*>0.05). HR/SpO_2_ increased more than 10% compared to baseline data in patients with failed HFNC treatment at 24 hours after extubation (1.06 vs.0.93 , *P*< 0.05) while there was no significant change in the ROX index at the same time (6.54 vs. 8.61, *P*>0.05) (Table 3 and Fig. 4-5).

**Table 3:** Changes in physiological parameters in patients with successful or failed weaning in the HFNC treatment group

	Failed n = 40						Successful n = 318				
	4 h before extubation	4 h after extubation	8–12 h later	20–24 h later	36–40 h	4 h before reintubation	4 h before extubation	4 h after extubation	8–12 h later	20–24 h later	36–40 h
Heart rate, mean (SD)	†89.67(16.07)	†94.62 (17.57)	†94.26 (18.59)	*†99.78 (14.64)	*†115.05 (9.92)	*99.18 (18.88)	83.01(13.42)	*87.65 (14.48)	*87.93 (14.79)	*85.33 (14.28)	85.01 (14.56)
Respiratory rate, mean (SD)	19.41(4.09)	*†22.83 (4.29)	*22.64 (5.31)	*†24.40 (5.69)	26.88 (8.09)	*24.47 (4.81)	19.31(4.29)	*21.21 (4.75)	*21.29 (4.80)	*21.00 (4.58)	*21.02 (4.91)
Tidal volume, mean (SD)	527.92(146.70)	–	–	–	–	–	504.81(126.12)	–	515.93 (132.49)	–	–
MBP, mean (SD)	81.10(13.91)	80.82 (15.61)	83.41 (14.24)	81.46 (14.29)	81.91 (12.14)	*83.97 (14.58)	78.34(11.79)	79.95 (12.62)	79.32 (14.04)	79.18 (12.32)	78.93 (11.57)
pH, mean (SD)	7.39(0.07)	7.37 (0.12)	7.40 (0.08)	7.36 (0.10)	7.42 (0.08)	7.38 (0.10)	7.40(0.06)	7.41 (0.07)	7.42 (0.06)	*7.43 (0.06)	*7.45 (0.06)
PaO_2,_ median [Q1, Q3]	89.25[83.50, 104.62]	91.50 [74.38,119.00]	*75.50 [64.50,95.00]	*81.00 [72.50,82.75]	75.50 [68.75,82.25]	*76.75 [66.75,98.50]	92.50[80.00, 110.00]	*83.00 [71.00,99.00]	*81.00 [71.00,97.00]	*78.00 [69.50,87.00]	*79.00 [68.75,104.50]
PaCO_2,_ mean (SD)	39.52(6.60)	41.78 (7.62)	39.61 (6.56)	39.00 (9.94)	47.50 (4.95)	40.09 (9.12)	40.00(6.83)	39.65 (7.36)	*37.82 (6.83)	*37.72 (8.68)	37.76 (8.35)
SpO2, median [Q1, Q3]	96.50[95.00, 98.29]	*95.12 [93.94,95.88]	*95.38 [93.44,96.43]	*94.25 [93.66,95.62]	95.00 [94.50,96.25]	*93.27 [91.69,95.29]	97.00[95.00, 98.50]	*95.00 [93.75,96.59]	*95.00 [93.50,96.50]	*95.00 [93.75,96.50]	*95.25 [93.55,96.94]
PaO_2_/FiO_2_, median [Q1,Q3]	208.00[170.00, 229.88]	*151.61 [133.75,171.50]	*129.00 [100.83,130.00]	*95.30 [85.99,111.55]	*†75.50 [68.75,82.25]	*98.84 [79.38,147.03]	201.29[163.20, 238.35]	*137.75 [102.12,175.50]	*126.08 [100.08,171.39]	*126.00 [90.31,171.00]	*133.53 [110.00,171.08]
ROX Index	†8.61 [7.57,9.45]	7.38 [5.27,9.49]	6.95 [5.25,8.75]	6.54 [4.82,9.33]	5.32 [3.93, 8.12]	*3.54 (0.43)	11.63 [9.22,13.51]	*7.29 [6.33,8.62]	*7.97 [7.08,9.01]	7.51 [6.97,8.06]	12.24 [9.55,14.94]
RSBI	40.35[30.71, 54.80]	–	–	–	–	*49.88 [40.31,55.09]	39.22[29.87, 50.51]	–	–	–	–
HR/PaO2	†0.97(0.22)	1.05 (0.37)	1.17 (0.34)	*1.25 (0.27)	1.64 (0.49)	*1.25 (0.45)	0.89(0.27)	*1.07 (0.33)	*1.05 (0.32)	*1.13 (0.58)	*1.09 (0.35)
HR/SpO2	†0.93(0.16)	†1.00 (0.19)	0.99 (0.19)	*†1.06 (0.16)	*†1.22 (0.10)	*1.06 (0.20)	0.86(0.14)	*0.92 (0.15)	*0.93 (0.16)	*0.90 (0.15)	*0.89 (0.15)

^†^*P* < 0.05 versus value of patients with successful weaning, *P < 0.05 vs value at baseline

**Fig. 6:**
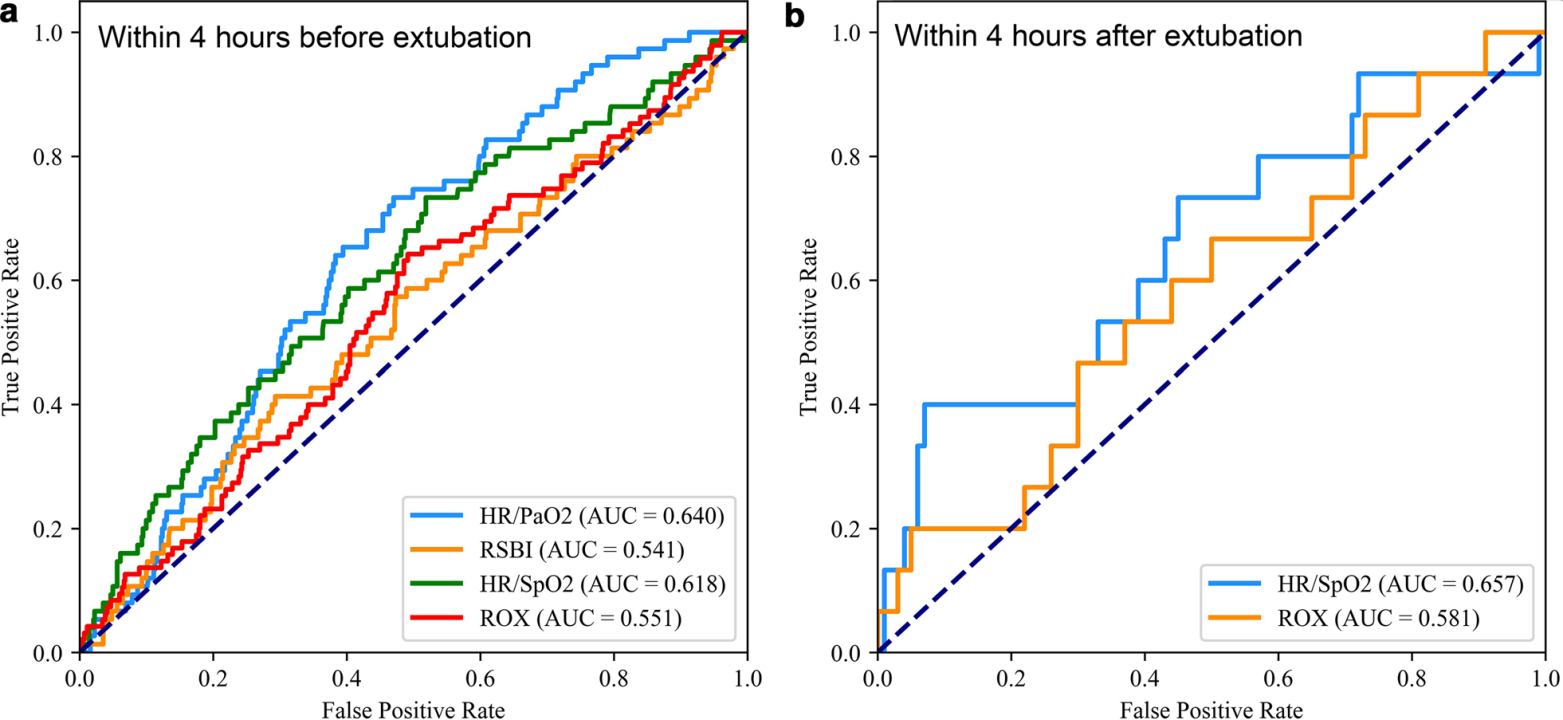
The ROC curves of HR/PaO_2_, HR/SpO_2_, the ROX index, and RSBI for 48-h reintubation prediction in the HFNC treatment group. **a** The ROC curves within 4 h before extubation; **b** the ROC curves within 4 h after extubation

According to values at 4 hours before extubation, the AUCs of HR/PaO_2_ and HR/SpO_2_ were 0.640 and 0.618 for predicting 48-hour reintubation, respectively, which were larger than that of RSBI (AUC=0.541) and ROX index (AUC=0.551). According to values at 4 hours after extubation, the AUC of HR/SpO_2_ were 0.657 for predicting 48-hour reintubation, which were larger than that of ROX index (AUC=0.583). The specificity reached 93% when the cut-off point of HR/SpO_2_ was 1.20 at 4 hours after extubation (Table 4 and Fig. 6).

**Table 4:** Predicting power of HFNC failure by HR/PaO_2_, HR/SpO_2_, RSBI and the ROX index at 4 h before and after extubation

	AUC (95% CI)	*P*	Cutoff value	Youden Index	Sensitivity	Specificity	PPV	NPV
4 h before extubation								
HR/PaO_2_	0.640 [0.584, 0.694]	*P* < 0.01	0.829	0.263	0.733	0.530	0.159	0.943
HR/SpO_2_	0.618 [0.551, 0.683]	*P* < 0.01	0.830	0.215	0.733	0.481	0.146	0.937
RSBI	0.541 [0.467, 0.607]	*P* < 0.01	48.4	0.120	0.413	0.707	0.146	0.909
ROX index	0.551 [0.488, 0.610]	*P* < 0.01	0.107	0.168	0.640	0.528	0.141	0.924
4 h after extubation								
HR/SpO_2_	0.657 [0.571, 0.724]	*P* < 0.01	1.203	0.330	0.400	0.930	0.462	0.911
ROX index	0.583 [0.519, 0.629]	*P* < 0.01	6.376	0.020	0.800	0.220	0.133	0.880

## Discussion

In our study, more than 500,000 medical records from 2008 to 2019 were selected from MIMIC-IV, and 801 patients with moderate to severe hypoxemia during mechanical ventilation weaning who received HFNC or NIV therapy were finally included. There were no significant differences in primary outcomes, including the 28-day reintubation rate and 28-day mortality, between the HFNC treatment group and the control group after PSM. Consistent results were confirmed in patients with moderate and severe hypoxemia. HFNCs can provide constant airflow and oxygen concentration with a small amount of positive end-expiratory pressure [11–13]. Therefore, the therapeutic effect of HFNC is better than that of conventional oxygen, including nasal catheters and facemasks [5, 14, 15]. Most research designs in recent years have been noninferior studies of HFNC and NIV, but the specific indication of hypoxemia is not clear. HFNC is noninferior to NIV for preventing postextubation respiratory failure in patients at high risk of reintubation or resolving acute respiratory failure in patients who receive cardiothoracic surgery. As the better tolerance with HFNC and a higher airway pressure delivered by NIV, combined treatment may be a better clinical option. Thille reported that the combined treatment could reduce the reintubation rate within 7 days compared to the use of HFNC alone [16]. In these studies, the mean oxygenation index of those patients with moderate hypoxemia was nearly 200 mmHg [3, 4]. Our study found that the effect of HFNC therapy was not inferior to that of NIV, even for severely hypoxemic patients with median oxygenation index of 170 mmHg.

The reintubation rate for ICU patients weaning from mechanical ventilation is approximately 10% [17], but it can reach 20% in patients at high risk when HFNC fails, and the timing of reintubation is mostly concentrated within 48 h after weaning [3, 4], which is consistent with our results. Therefore, patients who received reintubation within 48 h were regarded as having treatment failure in the HFNC treatment group, and we tried to predict reintubation within 48 h after extubation [18].

The longer length of ICU stay followed a longer duration before reintubation with the use of HFNC compared with NIV, which is in contrast to previous findings [5]. However, the mortality of patients who received reintubation within 48 h was not higher than that of patients who received reintubation 48 h after extubation in the HFNC group. In contrast to our findings, a previous study found that delayed intubation in patients with hypoxemia who received HFNC therapy might increase mortality [19]. The different results may be caused by different experimental designs and cohort sample sizes.

Although RSBI is routinely used as a clinical predictor of extubation failure, the threshold value for RSBI less than 105 had poor predictability for weaning success when measured at baseline during the spontaneous breathing trial, and it can be significantly affected by the level of ventilator support [20–22]. Moreover, the tidal volume is not routinely monitored after weaning. In patients with acute hypoxemic respiratory failure, the respiratory rate was a predictor of intubation under standard oxygen but not under high-flow nasal cannula oxygen or noninvasive ventilation [23]. Studies have shown that effective therapy for HFNC can decrease the work of breathing and reduce the respiratory rate of patients [24, 25]. Therefore, we think that the RSBI composed of tidal volume and respiratory rate is not a good predictor for reintubation with HFNC failure. ROX index is defined as the ratio of SpO_2_/FiO_2_ to respiratory rate [26], which needs further verification as a predictor of HFNC failure. At present, a simple and clear predictor for whether patients need early reintubation after weaning is still needed, and the timing of switching to invasive ventilator therapy is also not clear when HFNC fails [27, 28].

Respiratory work and oxygen consumption could be reduced with effective HFNC therapy. According to stroke volume × heart rate = cardiac output, heart rate decreased with cardiac output decreasing. And respiratory rate also decreased with less respiratory work. As feature importance was obtained by machine learning algorithm, we could infer that heart rate may be a more important and sensitive risk factor than respiratory rate. SpO_2_/FiO_2_ is a more accurate parameter to reflect oxygenation status than SpO_2_ according to basic physiology. But a predictor with two variables are obviously more simple and practical than the predictor with three variables. So we collected the two most important variables heart and SpO_2_ to form the predictor HR/SpO2 instead of the ratio of HR to SpO_2_/FiO_2_. Therefore, we propose to use HR/PaO_2_ or HR/SpO_2_ as predictors of reintubation.

As serial measurements of the RSBI and ROX index could more accurately predict successful weaning from mechanical ventilators [20, 29], we also observed the dynamic changes in these two indexes during extubation. The AUCs of HR/SpO_2_ according to values at 4 h before and after extubation to predict reintubation were larger than those of ROX index. The HR/SpO_2_ of patients with failed HFNC treatment was higher than that of patients with successful HFNC treatment within 4 h after weaning, but there was no significant difference of ROX index at the same time. Both HR/SpO_2_ and ROX index changed more than 10% compared to baseline data in patients with failed HFNC treatment at 24 h. The specificity of predicting HFNC treatment failure reached 93% when the threshold value of HR/SpO_2_ was 1.20 at 4 h after extubation, which was larger than that of ROX index. Therefore, HR/SpO_2_ may be a more sensitive and accurate predictor than ROX index for reintubation when HFNC treatment fails.

### Limitations

Our study is a retrospective study based on the MIMIC-IV database. The daily time of HFNC and NIV treatment in the treatment group and the control group was not extracted, which would have an impact on the treatment effect. Although most of high risk factors for reintubation were included and matched in the propensity score, there were few high risk factors not included because data missed in this retrospective study. Although the sample size was not small and propensity score matching ensured low heterogeneity in the included patients, the results of this study need to be verified by multicentre, large-sample prospective studies.

## Conclusions

The treatment effect of HFNC therapy is not inferior to that of NIV, even on patients with oxygenation index from 160 to 180 mmHg when weaning from ventilator. HR/SpO_2_ is more early and accurate in predicting HFNC failure than ROX index within 48 h after extubation.

## Data Availability

The datasets analysed during the current study are available in the MIMIC-IV repository, https://physionet.org/content/mimiciv/0.4/.

## Abbreviations

HFNC: High-flow nasal cannula
NIV: Noninvasive ventilation
MIMIC-IV: The medical information mart for intensive care IV
ICUs: Intensive care units
BMI: Body mass index
SAPS-II: The simplified acute physiology scoring II
ABG: Arterial blood gas
IQR: Interquartile range
PSM: Propensity score matching
XGBoost: The extreme gradient boosting
SHAP: The Shapley additive explanations
AUC: The area under the receiver operating characteristic curve
RSBI: The rapid shallow breathing index
ROX index: The ratio of SpO2/FiO2 to respiratory rate
