# Correction to: Efects of high-flow oxygen therapy on patients with hypoxemia after extubation and predictors of reintubation: a retrospective study based on the MIMIC-IV database

**DOI:** 10.1186/s12890-021-01562-y

**Published:** 2021-06-09

**Authors:** Taotao Liu, Qinyu Zhao, Bin Du

**Affiliations:** 1 Department of Surgical Intensive Care Unit, Beijing Hospital, National Center of Gerontology, Institute of Geriatric Medicine, Chinese Academy of Medical Sciences, Beijing, 100730 China; 2 College of Engineering and Computer Science, Australian National University, Canberra, 2600 Australia; 3 Department of Medical Intensive Care Unit, State Key Laboratory of Complex Severe and Rare Diseases, Peking Union Medical College Hospital, Chinese Academy of Medical Science and Peking Union Medical College, 1 Shuai Fu Yuan, Beijing, 100730 China

## Correction to: BMC Pulm Med (2021) 21:160 https://doi.org/10.1186/s12890-021-01526-2

Following publication of the article, the authors flagged that the article had published with incorrect affiliation details in the author list.

The article has since been corrected and the updated author list can be found in this correction.
